# Supplementary material for: A phenotypic small-molecule screen identifies halogenated salicylanilides as inhibitors of fungal morphogenesis, biofilm formation and host cell invasion
Source: Sci Rep. 2018 Aug 1;8:11559. doi: 10.1038/s41598-018-29973-8 (PMC6070544; doi:10.1038/s41598-018-29973-8)
Supplement: Supplementary file 1 — Table S1 [file 41598_2018_29973_MOESM1_ESM.docx]

A phenotypic small-molecule screen identifies halogenated salicylanilides as inhibitors of fungal morphogenesis, biofilm formation and host cell invasion

Carlos Garcia, Anaïs Burgain, Julien Chaillot, Émilie Pic, Inès Khemiri and Adnane Sellam

Supplementary Information

**Table S1.** Confirmation of microarray data by quantitative real-time PCR (qPCR). The reported values are the means ±STDV of two independent biological replicates

| **Gene** | **Fold-change** (±STDV) | |
| --- | --- | --- |
|  | **15 min** | **60 min** |
| *ICL1* | 524.52 ± 34.25 | 47.29 ± 5.78 |
| *MLS1* | 26.57 ± 0.39 | 3.69 ± 1.44 |
| *GDH2* | 32.07 ± 0.52 | -2.5 ± 0.31 |
| *MDR1* | 1.06 ± 0.38 | 2.48 ± 1.06 |
| *ERG25* | -10.48 ± 0.07 | -3.08 ± 0.31 |
| *ZRT2* | -22.55 ± 0.04 | -1.37 ± 0.49 |
| *LAC1* | -10.72 ± 0.04 | 1.08 ± 0.89 |
| *CDC47* | -8.31 ± 0.10 | -1.68 ± 0.16 |
